# Identification of Wnt/β-catenin modulated genes in the developing retina

**Published:** 2012-03-16

**Authors:** Andrew Ha, Carol Perez-Iratxeta, Hong Liu, Alan J. Mears, Valerie A. Wallace

**Affiliations:** 1 Vision Program, Ottawa Hospital Research Institute, Ottawa, Ontario, Canada; 2 Regenerative Medicine Program, Ottawa Hospital Research Institute, Ottawa, Ontario, Canada; 3 Department of Ophthalmology, University of Ottawa, Ottawa, Ontario, Canada; 4 Department of Biochemistry, Microbiology and Immunology, University of Ottawa, Ottawa, Ontario, Canada; 5 Department of Cellular and Molecular Medicine, University of Ottawa 451 Smyth Road, Ottawa, Ontario, Canada

## Abstract

**Purpose:**

During mammalian eye development, the restriction of Wnt/β-catenin signaling at the junction of the neural retina and the retinal pigment epithelium in the peripheral eyecup is required for the development of the ciliary margin, a non-neural region of the eyecup that is the precursor of the ciliary body and iris of the adult eye.

**Methods:**

To identify genes that are modulated by β-catenin activity in the embryonic retina, we performed gene expression profiling in Li^+^-treated retinal explants, a pharmacological model of β-catenin activation. The Li^+^-modulated gene data set was searched for β-catenin/T-cell specific transcription factor binding sites.

**Results:**

Functional annotations of this data set revealed significant enrichments for genes involved in chromatin organization, neurogenesis, and cell motion/migration. Quantitative real-time polymerase chain reaction (qRT–PCR) analysis confirmed the modulation of 12 genes in Li^+^-treated explants and retinas of mice with Cre-mediated induction of constitutively active β-catenin (β-cat^act^). In situ hybridization revealed β-catenin-specific upregulation of cyclin-dependent kinase inhibitor 1A (P21) [*Cdkn1a*] and tumor necrosis factor receptor superfamily, member 19 (*Tnfrsf19*) in the developing retina consistent with the antineurogenic and proliferation changes associated with ectopic Wnt/β-catenin signaling in the eyecup.

**Conclusions:**

This data set of Li^+^-modulated genes provides a valuable resource for characterizing the Wnt/ β-catenin regulated gene network in eyecup patterning.

## Introduction

The vertebrate eye comprises tissues originating from several embryonic lineages, including neuroectoderm, ectoderm, neural crest, and mesoderm. The coordinated development of these multiple tissue types is governed by intercellular interactions, which makes the eye an excellent model in which to study morphogenesis and inductive events during central nervous system development. At early stages of eye development, the neuroectoderm-derived optic vesicles invaginate to form a bilayered optic cup. The outer layer differentiates as the retinal pigment epithelium, and the inner layer is patterned into the central neural retina and peripheral ciliary margin (CM). The CM gives rise to the distal iris and proximal highly folded non-pigmented ciliary epithelium that forms the inner layer of the mature ciliary body (CB), which functions in secreting aqueous humor [1]. Notably, the CM does not undergo neurogenesis, despite the structure’s neuroectodermal origin. The development of this CB structure is of particular interest because aberrant development and function of the ciliary epithelium results in eye disease, most notably glaucoma [2] and because of the potential for retinal regeneration. The peripheral retina in the adult eye of lower vertebrates contains a source of stem cells that generate new retinal neurons throughout the life of the animal [3], and cells with clonogenic capacity can be isolated and cultured from the adult mammalian CB [4]. However, the in vivo potential of these cells to undergo neurogenesis in the mammalian retina has yet to be demonstrated.

The genetic and intercellular interactions that regulate early eyecup patterning and CB development are beginning to be understood. Although signals from the lens have been implicated in the induction of the CB [5,6], more recent studies suggest that the lens may be more involved in maintenance rather than induction of the CM [7]. With respect to the molecular regulation of CM/CB development, orthodenticle homolog 1 (*Otx1*) and LIM homeobox transcription factor 1 beta (*Lmx1b*) and bone morphogenetic protein signaling are all required for normal CM specification and differentiation [8–10]. More recently, canonical Wnt signaling has been implicated as a positive regulator of CM development [11,12].

The Wnt signaling pathway is a key regulator of patterning, growth, and cell fate in vertebrates [13]. Secreted Wnt ligands bind to Frizzled (Fzd) receptors and transduce intracellular signals through the canonical Wnt/β-catenin, planar cell polarity, and the Wnt/Ca2^+^ pathways [14]. In the canonical Wnt/β-catenin pathway, binding of a Wnt protein to Fzd results in the stabilization and accumulation of intracellular β-catenin, which then translocates to the nucleus where the protein interacts with the transcription factors T-cell specific transcription factor (TCF) and lymphoid enhancer-binding factor to transactivate target genes [13].

Several Wnt and Fzd receptors and other Wnt pathway components are expressed in the developing eye in several vertebrate species [15–17]. While the function of Wnt signaling in proliferation control appears to be species specific [18], there is a conserved requirement for Wnt/β-catenin signaling in regulating peripheral eye development. In *Xenopus*, canonical Wnt signaling in progenitor cells at the peripheral margin of the retina promotes neurogenesis [16]. Similarly, in zebrafish, chicks, and mice, Wnt expression and canonical Wnt signaling are active in the peripheral eyecup [12,15,17,19]. However, in contrast to *Xenopus*, the function of canonical Wnt signaling in the mouse and chick is to inhibit neurogenesis, and to promote the development of peripheral eye structures [11,12,17,19–21]. In the mouse and chick, Wnt2b is expressed in the RPE overlying the CM [15,17], and gain- and loss-of-function studies demonstrate that canonical Wnt pathway signaling is necessary and sufficient for CM and CB development at the expense of the neural retina [12,15,17].

Activation of Wnt signaling in the mouse retina was associated with increased expression of CM markers, including homeobox, msh-like 1 (*Msx1*), *Otx1*, and bone morphogenetic protein 4 (*Bmp4*), and downregulation of basic helix–loop–helix (bHLH) and homeodomain transcription factors and CyclinD1, which is consistent with the inhibitory effect of Wnt activation on neurogenesis and proliferation [11,12,20]. However, the exact molecular mechanism of β-catenin-mediated promotion of CM/CB development is poorly understood. To identify candidate Wnt/β-catenin target genes in CM development, we used microarrays to survey differentially expressed genes in Li^+^-treated retinal explants. Previously we showed that Li^+^ treatment, a well characterized activator of canonical Wnt signaling [22] in mouse embryonic retinal explants, mimics the CM-promoting effects of in vivo β-catenin signaling in that it inhibited proliferation and neurogenic gene expression and promoted CM gene expression [11]. To identify candidate β-catenin targets among the Li^+^-modulated genes, we searched the genomic sequences of these genes for conserved TCF consensus motifs. We validated the Wnt/β-catenin modulation of 12 genes in the retinal explants and retinas of mice with constitutive β-catenin activation in the peripheral retina (β-cat^act^). We show that the upregulation of two candidate target genes, cyclin-dependent kinase inhibitor 1A (P21) [*Cdkn1a*] and tumor necrosis factor receptor superfamily, member 19 (*Tnfrsf19*), is specific to β-catenin-activated regions of the embryonic mouse retina, suggesting that they may play a role in mediating the biologic effects of canonical Wnt signaling in the developing eye.

## Methods

### Explant culture

CD1 wild-type mice (obtained from Jackson Laboratories, Bar Harbor, ME) were time-mated to generate E14.5 embryos, with day 0 of gestation designated by the presence of the vaginal plug. The eyes were harvested from the embryos, and the RPE was removed in CO_2_-independent medium. The retina was then transferred to ethanol sterilized, 13 mm (0.8 μm pore-size) Nucleopore polycarbonate filters (Whatman, Piscataway, NJ) with the anterior lens oriented down onto the filter. The retinal explants were cultured at 8% CO_2_ and 37 °C in 500 µl serum-free retinal explant medium (1:1 F12/Dulbecco’s Modified Eagle’s Medium, transferrin [100 mg/ml], insulin [10 µg/ml], BSA [Fraction V: 100 mg/ml], putrescine [16 µg/ml], progesterone [60 ng/ml], sodium selenite [40 ng/ml], and gentamicin [25 µg/ml]). The Wnt/ β-catenin signaling pathway was activated by supplementing the retinal explant medium with 20 mM LiCl for 24 h. Control explants were treated with 20 mM NaCl.

### Microarray analysis

Gene expression profiling was performed on E14.5 retinal explants cultured in the presence of 20 mM NaCl (control) or 20 mM LiCl for 24 h, a time-period previously determined to be optimal for β-catenin activation by Li^+^ [11]. For each biologic replicate (NaCl or LiCl), RNA was isolated from pooled explants (eight explants/treatment group) using RNeasy mini kits (Qiagen, Valencia, CA) generating a total of 10–15 ug for each sample. RNA samples (targets) were labeled with Cy5 or Cy3 using 3DNA Array 900 kits (Genisphere, Hatfield, PA) following the manufacturer’s instructions. Li^+^ and control samples were then cohybridized to MEEBO 38.5K arrays (Microarrays Inc., Huntsville, AL). For each pairwise comparison, a dye-flip experiment was also performed (reversing which samples were labeled with Cy5 or Cy3) to correct for dye bias, for a total of four microarrays (two biologic and two technical replicates). Signals were quantified using the ScanArray express (Perkin Elmer, Waltham, MA), and intra-array normalization (Cy5/Cy3) was performed with LOWESS [23] using the ProScan Array Express software package (Perkin Elmer). M (log_2_ ratio of Li^+^/control signal) and A values (log_2_ average signal strength) were then determined for all probes. Differentially expressed genes were identified by comparing Li^+^ to control signals across the four arrays under the following criteria. A probe (gene) was scored as differentially expressed on an array if it showed at least a 1.5-fold change. To score a probe as differentially expressed in the experiment, the following criteria had to be met: If a probe was differentially expressed in 3/4 arrays, all three must display a minimum twofold change (i.e., 2/2/2/0). If a probe was differentially expressed in all four arrays, a minimum of a 1.8 fold change had to be observed in at least three of the arrays (1.8/1.8/1.8/1.5), or a twofold change in at least two of the arrays (2.0/2.0/1.5/1.5). In addition, a probe must also display a sufficient signal with an average A minimum value of 7 (log_2_); otherwise, the probe was considered to have too low a signal to be reliably measured (i.e., low/absent expression).

### Gene annotation and motif searches

Gene Ontology (GO) analysis was performed for all differentially expressed genes using the online tool DAVID Bioinformatics Resources 6.7. Biologic processes GO terms annotating these genes were classified into major categories and tested for significant enrichment. To prioritize the list of candidate genes for potential Wnt/β-catenin targets, the microarray results were mined for genes that contain a consensus T-cell factor (TCF) binding site. Consensus TCF-binding sites (CCTTTGWW) [24] are highly conserved motifs generally clustered in groups of one to four binding sites upstream from the transcriptional start site [25–27]. Therefore, an interval of 5 kb upstream of the transcriptional start site to 1 kb downstream of the transcriptional stop site was used to screen the genomic sequence (release mm8) of differentially expressed genes in Li^+^-treated retinal explants for genes containing TCF-binding site(s). This analysis did not significantly reduce the number of differentially expressed genes as the consensus TCF-binding site occurs by chance approximately once every 16 kb in the genome. To refine the list of candidate genes, the results were filtered for genes that contain at least one conserved TCF-binding site. To define conservation, we used the conservation scores phastCons [28] available at the University of California, Santa Cruz (UCSC) Genome Bioinformatics [29]. These conservation scores are generated from a multiple alignment of 16 vertebrate genomes to the mouse genome [30,31]. For each match, we computed the average conservation score. Genes with TCF matches that had an average score greater than 0.7 (out of a possible 1) were identified as potential Wnt targets.

### RNA extraction, cDNA synthesis, and quantitative real-time–polymerase chain reaction

Pools of two or three retinal explants were placed in 1 ml of TRIzol and sonicated with five pulses (8–10 s each) at an amplitude of 35% followed by RNA extraction as per manufacturer’s guidelines (Invitrogen/Life Technologies, Carlsbad, CA). cDNA was synthesized from 2 µg of total RNA using M-MLV reverse transcriptase (Invitrogen/Life Technologies, Carlsbad, CA) following the manufacturer’s protocols. Quantitative real-time–polymerase chain reaction (q-RT–PCR) was performed using 1 μl of cDNA with JumpStart qPCR mastermix (Sigma-Aldrich, St. Louis, MO) following manufacturer’s guidelines but in a 25 μl reaction volume. Gene-specific primer pairs (Table 1) were designed using Primer3 software, with 100% homology to the target sequence and confirmed using a BLAST search (National Center for Biotechnology) [32]. The primers were designed with a melting temperature of 58–59 °C, and the amplicons ranged from 100 to 200 bp. The PCR reactions were performed in triplicate using a Stratagene Mx3000P (Agilent Technologies, Inc., Santa Clara, CA) with the following cycling parameters: 94 °C for 3 min, followed by 40 cycles of 94 °C for 30 s, annealing at 58 °C for 30 s, and extension at 72 °C for 1 min. Changes in gene expression were quantified using the 2^-ΔΔCT^ method with normalization to the housekeeping genes 18S and glyceraldehyde-3-phosphate dehydrogenase (*Gapdh*). Statistical significance was evaluated using a two-tailed, unpaired Student *t* test, and a p≤0.05 was considered statistically significant.

**Table 1:** QPCR primer sets

**Gene**	**Sequence (5′-3′)**
*Otx1*	F: CGGGAATGGAACGAAAAC
	R: GCTCGTCTCCGAACCCGA
*Msx1*	F: CACCGCAACCGCCAT
	R: TGCCCAAGTGCTGCAC
*Cdkn1a*	F: AGAGTGCAAGACAGCGACAA
	R: GTCCAATCCTGGTGATGTCC
*Med12*	F: TATACCGGCAGCAGCAAC
	R: GGAAGAACTAGGGGTCATCTG
*Wif1*	F: TAAGAGGTATGGAGCCAGCC
	R: ATCCCTTCTATCCTCAGCCTT
*Efna3*	F: GTGAAGATCAACGTGTTGGAA
	R: GAGGCCAAGAGCTGCAT
*Axin2*	F: AGACCGGTCACAGGAGTG
	R: CAGGCAGACTCCAATGGGTA
*Cdc25b*	F: ATCCTCAAGAGGCTAGAGCG
	R: ACGGGCCTTAGGTTCTTCA
*Epha2*	F: TGATCCCCGAGTGTCCA
	R: CAGATAGGAATCCCCACTGTGT
*Tnfrsf19*	F: GAGAAGTACCAATTCCCTCAA
	R: AGATGCTGCGCTTTCGT
*Klf10*	F: TTGAGACAGTCCCAGCATTTG
	R: GGCAGCATCGGAGAAAGAT
*Lix1*	F: GCCCTGGACTGGATTATGAA
	R: CGTGAGGCTTAGCTGGTCAG
*Ndrg2*	F: ACAAGTTAACTGGCCTTACGTCTT
	R: TGCGTGTTGGATGATACCTCT
*Prickle1*	F: TTGAAGAGAGGGGATCCAG
	R: ATGGGCATACTGGCTATAGAGGTT
*Serpine2*	F: CTTCATGTCTCTCACATCTTGC
	R: TACTATAAACCAGGGAGGTGATGA
*Syt13*	F: TTAAGTTCCCGGACATCTATGGT
	R: GACTCCTCTGTGGTCTCCAA

### In situ hybridization and X-gal staining

In situ hybridization (ISH) of cryosections of embryonic eyes was performed as previously described [33] using DIG-labeled antisense probes for *CyclinD1* (a kind gift from Gordon Peters, London Research Institute, London, UK) and *Msx1* (a kind gift from Yi-Hsin Liu, Keck School of Medicine, University of Southern California, Los Angeles, CA). All other riboprobes used in this study were generated by PCR amplification of cDNA from embryonic retinas. Briefly, gene-specific primer sets (Table 2) were used to produce 500–800 bp amplicons that were subcloned into the pGEM^®^-T Easy Vector (Promega, Madison, WI). The construct (insert + vector) was then sequenced to confirm insertion and orientation of the amplicon and antisense probes were synthesized from the linearized vector using T7 or SP6 RNA polymerase. 5-bromo-4-chloro-indolyl-β-D-galactopyranoside (X-gal) staining in cryosections of embryonic eyes was performed as previously described [15]. Briefly, embryonic tissue was fixed in 4% paraformaldehyde for 15 min before embedding for cryosectioning. Cryosections were cut at 12 µm and dried at room temperature for 2–6 h. Sections were immersed in Dulbecco’s Phosphate Buffered Saline with calcium and magnesium (PBS; Thermo Fisher Scientific, Waltham, MA) for 5 min and incubated in β-galactosidase (LacZ) staining buffer overnight at room temperature in the dark. Sections were then washed with PBS and mounted using glycerol/PBS (1:1). Images were analyzed using a Zeiss Axioplan 2 microscope and captured with an Axiocam camera (Carl Zeiss Canada Ltd, Toronto, ON, Canada) at 20× (0.8 N.A). Images were processed with Adobe Photoshop® CS2.

**Table 2:** Primer sets for ISH probes

**Gene**	**Sequence (5′-3′)**
*Cdkn1a*	F: CGGTGGAACTTTGACTTCGT
	R: CAGGGCAGAGGAAGTACTGG
*Tnfrsf19*	F: GAGACCCACCTCCGTCCTAC
	R: GGAGTCCTTGGAGCATCCTG
*Syt13*	F: AGTTGAGGATGTCTGTGTCAT
	R: ACCTTGACAGACACATCCTT
*Wif1*	F: CAAGTTGGTTCCCGTGTCT
	R: TTAAGTGAAGGCGTGTGTCG

### Transgenic mice

Transgenic mice were maintained and crossed as previously described [11]. The *α-Cre* (obtained from P. Gruss, Max-Planck Institute of Biophysical Chemistry, Goettingen, Germany [34]) and *Catnb^+/lox(ex3)^* [35] transgenic mouse lines were maintained on a C57BL/6 background, and the *TCF/Lef-LacZ* mouse line (obtained from D.Dufort, McGill University, Montreal, QC, Canada [36]) was maintained on a CD1 background. The *α-Cre* mice were crossed with the *TCF/Lef-LacZ* mice to create the *α-Cre;TCF/Lef-LacZ* mouse line. Heterozygous *α-Cre;TCF/Lef-LacZ* mice were crossed with the *Catnb^+/lox(ex3)^* mice to generate *α-Cre;Catnb^+/lox(ex3)^* or *α-Cre;Catnb^+/lox(ex3)^;TCF/Lef-LacZ* genotypes and were designated as β-cat^act^ mutant mice. Littermates with *α-Cre;Catnb^+/+^* or *α-Cre;Catnb^+/+^;TCF/Lef-LacZ* genotypes were designated as control. Genotyping for the transgenic mice was performed with PCR using the following primer pairs: α-Cre- (F) 5′-ATG CTT CTG TCC GTT TGC CG-3′ and (R) 5′-CCT GTT TTG CAG GTT CAG CG-3′; TCF/Lef-LacZ (F) 5′-CAG TGG CGT CTG GCG GAA AAC CTC-3′ AND (R) 5′-AAA CAG GCG GCA GTA AGG CGG TCG G-3′; Catnb+/lox(ex3) (F) 5′-GAC ACC GCT GCG TGG ACA ATG-3′ and (R) 5′-GTG GCT GAC AGC AGC TTT TCT G-3′. For analysis, E14.5 embryo heads were fixed in 4% paraformaldehyde phosphate buffer overnight and washed in PBS. The heads were then transferred to a 30% sucrose/PBS solution overnight and embedded in 1:1 optimal cutting temperature/ 30% sucrose/PBS mixture for cryosectioning.

## Results

### Gene expression profiling for Li^+^-modulated genes in retinal explants

**Figure 1:**
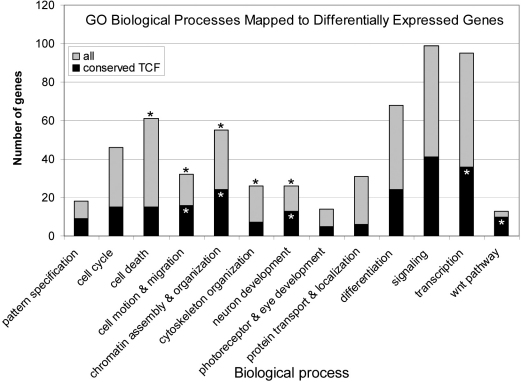
Graph indicates the frequency of annotated Gene Ontology (GO) terms to the non-redundant data set of all differentially expressed genes *and* those with conserved TCF binding sites. The asterisks indicate that the mapped term is over-represented in the data set based on an enrichment score of greater than 1 with GO Annotation clustering (DAVID).

To obtain a comprehensive profile of β-catenin-dependent gene expression in the retina, we performed microarray analysis on control and Li^+^-treated retinal explants. A total of 919 differentially expressed probes were identified in the Li^+^-treated explants, corresponding to 829 different genes, of which 386 were upregulated, 441 downregulated (Appendix 1), and two (engulfment and cell motility 1, ced-12 homolog [*Elmo1*] and uracil DNA glycosylase [*Ung*]) were reported as being both upregulated and downregulated. In the case of these last two genes, this observation may be explained in both instances by the differential response of alternative transcripts for these genes (probes detect different transcripts). Included in the list of upregulated genes were the CB markers *Msx1* and *Otx1*, which we have shown previously to be modulated by increased β-catenin activity [11] as well as known Wnt target genes, *Axin2* and Wnt inhibitory factor 1 (*Wif1*; The Wnt Homepage) [14]. Thus, the altered expression of known Wnt targets and CM markers is consistent with Li^+^ treatment mimicking a canonical Wnt signaling response in retinal explants. GO analysis of the differentially expressed genes in terms of their associated biologic processes identified a broad range of functional classes: 278 of the genes had no associated term, 336 genes accounted for 584 hits to 13 major categories, and the remaining 215 were associated with other biologic processes. There was significant enrichment (over-representation) of genes associated with chromatin assembly and organization, cell death, cell motion and migration, cytoskeleton organization, and neuron development (Figure 1).

**Figure 2:**
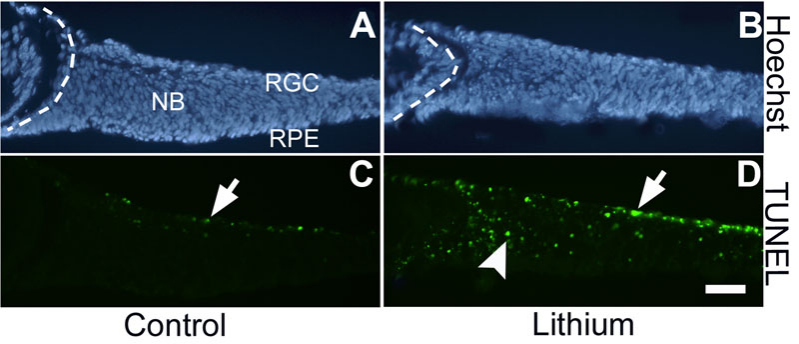
Reduced cell survival in Li^+^-treated explants. **A**-**D**: Nuclear (**A**, **B**) and TUNEL staining (**C**, **D**) in E14.5 retinal explants cultured in control (**A**, **C**) and Li^+^ (**B**, **D**) containing medium for one day. Cell death (indicated by TUNEL+ cells) is increased in the ganglion cell layer (arrow in **D**) and in the neuroblast layer (arrowhead in **D**). RGC, retinal ganglion cell layer; RPE, retinal pigment epithelium; NB, neuroblast layer.

The enrichment of cell death–associated genes in the Li^+^-upregulated data set raised the possibility that Li^+^ treatment had an impact on cell survival in retinal explants. To address this issue, we stained control and Li^+^-treated explants after 24 h with terminal deoxynucleotidyl transferase 2’-Deoxyuridine, 5′-Triphosphate (dUTP) nick end labeling to visualize apoptotic cells, which revealed a marked increase in dying cells in Li^+^-treated explants compared with controls (Figure 2). Thus, short-term Li^+^ treatment in explants is associated with an increase in cell death, which is consistent with the upregulation of cell death–specific genes in the microarray data set. However, we think this effect of Li^+^ is secondary because increased β-catenin activity in vivo is not associated with increased death [11] and because there was no significant enrichment for apoptotic genes after the data set was filtered for genes with conserved TCF binding sites (see below).

### Computational screen and target validation of candidate Wnt/β-catenin target genes

Wnt/β-catenin/TCF signaling regulates transcription through the interaction of β-catenin/TCF with TCF/Lef-1 consensus binding motifs in promoters of target genes. Consensus TCF-binding sites (CCTTTGWW [24]) are highly conserved and are generally clustered in groups of one to four binding sites located up to 1 kb upstream from the transcriptional start site [25–27]. Therefore, to identify putative TCF/Lef-1 target genes we filtered the list of Li^+^-modulated genes for genes that contained a TCF binding site in an interval spanning 5 kb upstream of the transcriptional start site to 1 kb downstream of the transcriptional stop site. This analysis reduced the number of differentially expressed genes from 829 to 683. The list was further refined by filtering for genes that contain a TCF-binding site with an average conservation value higher than 0.7 out of a possible 1 (see Methods). This final screen reduced the number of candidate targets to 225 genes with 84 up, 139 down, and two reported as both up and down. The final list includes general Wnt target genes *Wif1* and *Axin2* and Wnt modulated genes in the CB, *Msx1* and *Otx1* [11], confirming the utility of this approach to capture Wnt/TCF targets (Appendix 2). GO annotation revealed a significant enrichment for genes associated with chromatin assembly and organization, cell motion and migration, neuron development, and Wnt signaling and transcription, with the latter two categories showing an increase in representation in this final group (Figure 1). In most GO categories, the number of genes upregulated versus downregulated were relatively equivalent; however, this was not the case with genes associated with neuron development (11 down, two up), differentiation (16 down, eight up), or chromatin organization (23 down, one up).

**Figure 3:**
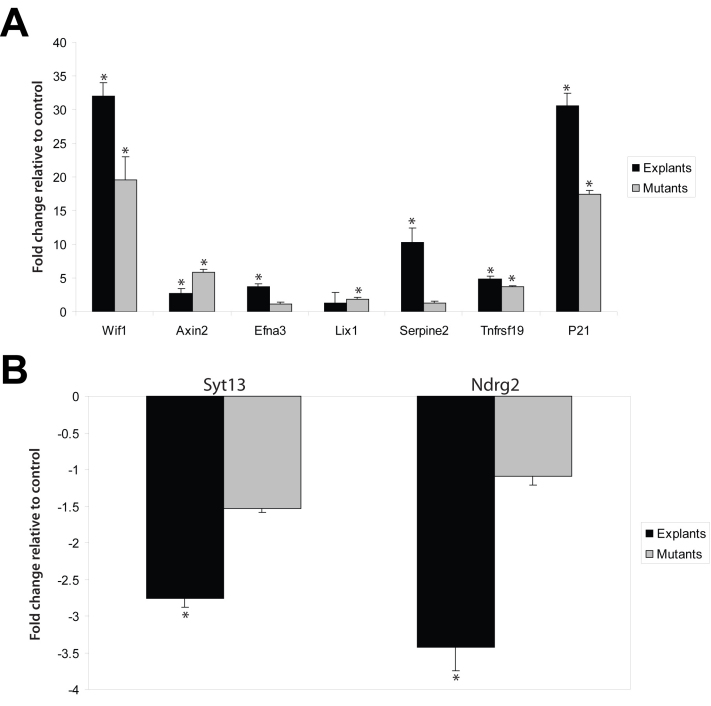
QRT–PCR analysis in Li+-treated retinal explants and in β-cat^Act^ mutant eyes of gene expression. Validation of upregulated (**A**) and downregulated (**B**) genes from the Li+-modulated data set. A total of three independent experiments were performed in triplicate (n=3). Data are normalized to *Gapdh* and 18S and presented as fold-change relative to control explant cultures and wild-type littermates±SD *p<0.05.

Increased β-catenin activity in the embryonic mouse retina alters neurogenesis and proliferation [11,12,20]. Therefore, genes with these functions were prioritized for validation (Table 3). Also included in this list for validation were genes from the unfiltered data set that exhibited a high fold-change in response to Li^+^, known Wnt targets in other tissues, or genes with several non-conserved TCF-binding sites (Table 3). We monitored gene expression with qRT–PCR in two gain-of-function models of β-catenin activity: Li^+^-treated E14 retina explants and whole E14 retinas of mice expressing a Cre-dependent constitutively active allele of β-catenin in the peripheral retina (β-cat^Act^; described in [11]). Consistent with the microarray analysis, the expression of *Wif1*, *Axin2*, ephrin A3 (*Efna3*), serine (or cysteine) peptidase inhibitor, clade E, member 2 (*Serpine2*), *Tnfrsf19*, and *Cdkn1a* was upregulated (Figure 3A and Table 3) and that of synaptotagmin XIII (*Syt13*), prickle homolog 1 (*Prickle1*), and N-myc downstream regulated gene 2 (*Ndrg2*) was downregulated in Li^+^-treated explants (Figure 3B and Table 3). A similar pattern of gene expression was also observed the retinas of β-cat^Act^ mice with the exception that expression of *Serpine2* and *Efna3* was unchanged and limb expression 1 homolog (*Lix1*) was increased, compared with Li+-treated explants (Figure 3 and Table 3). One explanation for differences in gene expression between explants and the β-cat^Act^ retina may be reduced sensitivity in the latter, because the retina samples contained a mixture of wild-type (central retina) and peripheral (mutant) tissue.

**Table 3:** Summary of candidate Wnt target gene validation.

				**qPCR validation**		
**Gene**	**Fold-change on microarray**	**# TCF binding sites**	**# conserved sites**	**Explants**	**β-cat^Act^**	**in situ β-cat^Act^**
**Wnt pathway**						
*Wif1*	29.2	7	1	+	+	+
*Axin2*	2.8	6	4	+	+	nd
**Ciliary margin markers**						
*Msx1*	2.3	6	3	nd	nd	+
*Otx1*	1.8	9	7	nd	nd	+
**Neuron development**						
*Epha2*	8.4	4	1	**-**	**-**	nd
*Efna3*	1.7	4	1	**+**	**-**	nd
*Syt13*	−11	6	0	**+**	**-**	+
*Prickle1*	−6	9	1	**+**	**-**	nd
*Ndrg2*	−2.8	4	2	**+**	**-**	nd
**Cell cycle**						
*Cdkn1a*	21.9*	3	0	+	+	+
**Differentiation**						
*Serpine2*	25.1	1	1	+	-	nd
*Klf10*	2.3	1	0	+	-	nd
*Cdc25b*	1.9	4	1	+	-	nd
*Lix1*	2.5**	12	4	-	+	-
**Signaling**						
*Tnfrsf19*	20.9	10	2	+	+	+
*Med12*	2	8	0	-	-	-

Gene expression was compared by q-RT–PCR analysis in [1] Li+-treated and untreated retinal explants [2]; whole retinas of E14 β-cat^Act^ and control littermates and by in situ hybridization in retina sections from β-cat^Act^ and control littermates at E14. (+) indicates that the expression of the gene exhibited the same trend (either up or down) as reported in the microarray analysis. (-) no change in the gene expression was detected or the change in expression was opposite to that reported in the microarray analysis. nd, not done. Asterisks indicate that the reported fold-change is the average value obtained from two (*) or three (**) different probes on the microarray.

**Figure 4:**
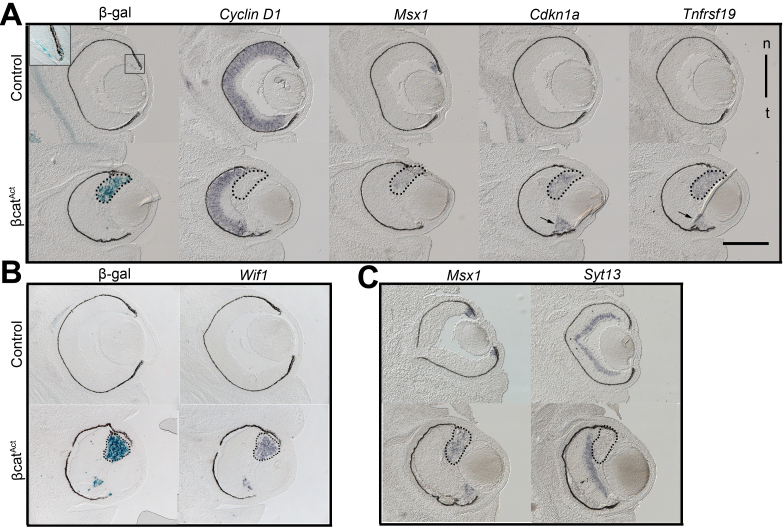
ISH analyses of target gene expression in the context of in ectopic β-catenin activation in vivo. **A**-**C**: Serial cryosections of Control (Cntr) and in β-cat^Act^ retinas at E14.5 were analyzed for expression of the indicated genes by ISH or for expression of the Tcf-LacZ reporter transgene with X-gal staining. The dotted outlines indicate the expanded CM region in the β-cat^Act^ retina, which is marked by X-gal staining (**A**, **B**) or expansion of *Msx1* expression (**A**, **C**). Each lettered image grouping represents a separate serial series. Sections were cut in the horizontal plane. n/t indicates the nasal/temporal orientation of the eyes in all images. Note that X-gal staining for the Tcf-reporter in wild-type mice is lower than previously reported, because the tissue was fixed overnight.

Next, we addressed how specific these changes in gene expression were to the region of the retina with active β-catenin signaling by performing in situ hybridization on retinal sections from β-cat^Act^ mutant mice. In β-cat^Act^ retinas, peripherally restricted expression of constitutively active β-catenin increases TCF-LacZ reporter activity (Figure 4) and expands the CM, as shown by the expansion of *Msx1* expression, a marker of the ciliary margin (Figure 4A,C and [11]). The expression of *Cdkn1a*, *Tnfrsf19*, and *Wif1* was increased and restricted to the expanded CM in the mutant eye, as their expression overlapped with the *Msx1* and Tcf-reporter expression domains and was excluded from the neural retina, which is marked by *CyclinD1* expression (Figure 4A,B). *Syt13* was expressed in the neural retina but not the expanded CM in the retinas of mutant mice (Figure 4C), which is consistent with the downregulation of this gene in the microarray data set (Table 1 and Table 3) and with the inhibition of neurogenesis that is a feature of β-catenin activation in this region of the developing eye [11]. ISH for *Lix1* and mediator of RNA polymerase II transcription, subunit 12 homolog (*Med12*) revealed that the former is undetectable in the retina and the latter is ubiquitously expressed, which is consistent with why these genes were not validated in the qRT–PCR analysis of the β-cat^Act^ retina (data not shown).

## Discussion

To identify candidate β-catenin target genes in the developing retina, we performed microarray expression profiling of Li^+^-treated retinal explants from E14 mice. To enrich for putative direct target genes, the data set was filtered to identify genes with conserved TCF binding sites. The final list of differentially expressed genes included known Wnt target genes and previously identified CM markers. Using in vitro and in vivo gain-of-function models, we confirmed the Li^+^/β-catenin modulation of 12 genes and demonstrated that the expression of two of these genes was enriched in the region of enhanced β-catenin activity in vivo. This approach thus has identified several interesting candidate genes for functional studies of the molecular mechanism of Wnt/ β-catenin-mediated patterning of the eyecup.

In previous studies, we showed that in vitro treatment with Li^+^ and in vivo transgenic activation of β-catenin activity induced markedly similar effects on gene expression, Wnt reporter gene induction, and proliferation [11]. However, we observed that Li^+^ increased cell death in explants, an effect that does not appear to be mimicked by β-catenin activation in vivo. This observation could reflect stage-specific differences in the response to β-catenin activity or indicate β-catenin-independent effects of Li+. Although it is well established that Li^+^-induced GSK3 inhibition activates the canonical Wnt signaling pathway [22,37,38], there are off-target effects of Li^+^ [reviewed by 39] as well as effects on other GSK3 substrates, such as p53, activator protein 1, and nuclear factor-κB [40–43]. As a way to identify the β-catenin-dependent subset of Li^+^-modulated genes, we filtered the data set for genes with conserved TCF binding sites. This list of candidate direct β-catenin target genes included known Wnt target genes and ciliary margin-restricted genes that have been shown to be modulated by β-catenin activity in the mouse and chick retina in vivo [11,12,44] suggesting that gene expression analysis of Li^+^-treated explants was enriched for genes that could be relevant to the impact of β-catenin activity at earlier stages in development.

Recently, several novel ciliary margin restricted and β-catenin inducible genes have been identified in the developing eyecup [44,45]. Only two genes in our Li^+^-modulated data set, *Otx1* and serum/glucocorticoid regulated kinase 1 (*Sgk1*), are represented in the Trimarchi et al. [44] study; however, differences in the screening approach likely account for the lack of overlap with the two approaches. Although inhibitor of DNA binding 3 (*Id3*) has been shown to be a direct target of β-catenin in the peripheral eye [45], we did not identify it as a Li^+^-modulated gene in our study likely because expression of the gene is not restricted to the CM at E14, the developmental stage of our screen.

Here we identify *Tnfrsf19* and *Cdkn1a* as candidate β-catenin responsive genes in the peripheral eyecup. The expression of both genes was upregulated in Li^+^-treated retinal explants and in the region of the peripheral retina with active β-catenin signaling in the β-cat^Act^ mice. However, we were unable to detect expression of these genes in the wild-type ciliary margin, possibly because their expression is normally too low to be detected by in situ hybridization, even at different developmental stages (Ha, unpublished). *Tnfrsf19* encodes a member of the tumor necrosis factor receptor superfamily of proteins that are involved in apoptosis, differentiation, and proliferation [46]. *Tnfrsf19* is expressed in embryonic epithelia [47–50] and postnatally in the hair follicle and neurons [47] and loss-of-function studies indicate a role for *Tnfrsf19* in hair follicle development [49] and axon regeneration [51]. In addition, *Tnfrsf19* is a direct target of Wnt signaling in somitogenesis. Although no eye phenotype has been described in the *Tnfrsf19^−/−^* mouse, Wnt regulation in somitogenesis [52] and expression in neural progenitors [53] make this gene an excellent candidate for future functional studies.

*Cdkn1a* is known as a repressor of cell cycle progression by inhibiting cyclin-dependent kinases. Although *Cdkn1a* is generally known as a member of the p53-dependent damage response pathway [54], this gene has also been found to play a role in development in which *Cdkn1a* is expressed in differentiating melanocytes [55] as well as acting as a positive cofactor of microphthalmia-associated transcription factor expression in melanoma cells [56]. Loss-of-function studies in *Cdkn1a^−/−^* mice have shown that *Cdkn1a* represses neuronal proliferation in the subgranular zone of the dentate gyrus of the hippocampus, suggesting that the gene functions to restrain neurogenesis in this region [57]. *Cdkn1a* activity also regulates the proliferative capacity of neural stem cells in the mammalian brain with loss of *Cdkn1a* function resulting in a reduced neural stem cell population in the forebrain of adult *Cdkn1a*^−/−^ mice [58]. Although *Cdkn1a* expression was not detected in the eyes of wild-type mice in this and previous studies [59], *Cdkn1a* has been shown to control patterning in development of the RPE [59]. *Cdkn1a*’s ability to arrest cell cycle progression makes this gene a possible candidate in Wnt-mediated CM development.

Activation of canonical Wnt signaling in the chick and mouse retina is associated with a slowing of the cell cycle, inhibition of neurogenesis, and conversion to CM/ciliary body [11,12]. Consistent with this complex response, the Li+-modulated data set included cell cycle regulators, particularly cell cycle inhibitors, and was enriched for genes involved in neuronal differentiation. Interestingly, the data set was also enriched for genes involved in cell migration and chromatin assembly and modification, highlighting two new avenues for future studies on the impact of β-catenin activity on cell behavior in the developing eyecup.

## **Appendix 1.** All differentially expressed probes.

To access the data, click or select the words “Appendix 1.” This will initiate the download of a Microsoft Excel (.xls) file. All probes determined to be differentially expressed by the experimental threshold criteria are shown with their associated Gene Ontology Biologic Process terms. Index refers to the microarray spot position. Fold-change is indicated for the response of the Li^+^ treated samples versus controls as an average value across all four arrays. Signal strength (A) is shown in log_2_ scale and is calculated as the average across all four arrays with 7 considered as the minimum threshold for which the target transcript is considered detectable. Gene Ontology terms that are associated with the gene are indicated by the green boxes.

## **Appendix 2.** All differentially expressed genes with conserved TCF binding sites

To access the data, click or select the words “Appendix 2.” This will initiate the download of a Microsoft Excel (.xls) file. All genes determined to be differentially expressed by the experimental threshold criteria and to have conserved TCF binding sites are shown with their associated Gene Ontology Biologic Process terms. Index refers to the microarray spot position. Fold-change is indicated for the response of the Li^+^ treated samples versus controls as an average value across all four arrays. Signal strength (A) is shown in log_2_ scale and is calculated as the average across all four arrays with 7 considered as the minimum threshold for which the target transcript is considered detectable. The number of conserved TCF sites is indicated. Gene Ontology terms that are associated with the gene are indicated by the green boxes.
